# Cost-effectiveness of ceftolozane/tazobactam plus metronidazole versus piperacillin/tazobactam as initial empiric therapy for the treatment of complicated intra-abdominal infections based on pathogen distributions drawn from national surveillance data in the United States

**DOI:** 10.1186/s13756-017-0264-2

**Published:** 2017-10-27

**Authors:** Vimalanand S. Prabhu, Joseph S. Solomkin, Goran Medic, Jason Foo, Rebekah H. Borse, Teresa Kauf, Benjamin Miller, Shuvayu S. Sen, Anirban Basu

**Affiliations:** 1 Merck & Co., Inc., Kenilworth, NJ USA; 2 University of Cincinnati College of Medicine, Cincinnati, OH USA; 3 Mapi Group, Houten, The Netherlands; 4 Baxalta US Inc., Boston, MA USA; 5 Shire, Lexington, MA USA; 6 Pharmaceutical Outcomes Research and Policy Program, University of Washington, Seattle, WA USA; 7 Center for Observational and Real World Evidence (CORE), Merck & Co., Inc., 2000 Galloping Hill Road, Kenilworth, NJ 07033 USA

**Keywords:** Cost-effectiveness analysis, Ceftolozane, Piperacillin, Tazobactam, Intraabdominal infections, United States, Drug resistance

## Abstract

**Background:**

The prevalence of antimicrobial resistance among gram-negative pathogens in complicated intra-abdominal infections (cIAIs) has increased. In the absence of timely information on the infecting pathogens and their susceptibilities, local or regional epidemiology may guide initial empirical therapy and reduce treatment failure, length of stay and mortality. The objective of this study was to assess the cost-effectiveness of ceftolozane/tazobactam + metronidazole compared with piperacillin/tazobactam in the treatment of hospitalized US patients with cIAI at risk of infection with resistant pathogens.

**Methods:**

We used a decision-analytic Monte Carlo simulation model to compare the costs and quality-adjusted life years (QALYs) of persons infected with nosocomial gram-negative cIAI treated empirically with either ceftolozane/tazobactam + metronidazole or piperacillin/tazobactam. Pathogen isolates were randomly drawn from the Program to Assess Ceftolozane/Tazobactam Susceptibility (PACTS) database, a surveillance database of non-duplicate bacterial isolates collected from patients with cIAIs in medical centers in the USA from 2011 to 2013. Susceptibility to initial therapy was based on the measured susceptibilities reported in the PACTS database determined using standard broth micro-dilution methods as described by the Clinical and Laboratory Standards Institute (CLSI).

**Results:**

Our model results, with baseline resistance levels from the PACTS database, indicated that ceftolozane/tazobactam + metronidazole dominated piperacillin/tazobactam, with lower costs ($44,226/patient vs. $44,811/patient respectively) and higher QALYs (12.85/patient vs. 12.70/patient, respectively). Ceftolozane/tazobactam + metronidazole remained the dominant choice in one-way and probabilistic sensitivity analyses.

**Conclusions:**

Based on surveillance data, ceftolozane/tazobactam is more likely to be an appropriate empiric therapy for cIAI in the US. Results from a decision-analytic simulation model indicate that use of ceftolozane/tazobactam + metronidazole would result in cost savings and improves QALYs, compared with piperacillin/tazobactam.

**Electronic supplementary material:**

The online version of this article (10.1186/s13756-017-0264-2) contains supplementary material, which is available to authorized users.

## Background

Intra-abdominal infections (IAIs) represent a wide variety of pathological conditions caused by inflammation or perforation of the intra-abdominal organs. In the latter case, complicated IAIs (cIAIs) arise causing localized or diffuse peritonitis [1]. Gram-negative pathogens, including resistant pathogens are responsible for over 70% of cIAIs [2]. Patients at a higher risk of treatment failure due to a resistant infection include those with health care-associated infection or prior antibiotic exposure [3]. Studies have shown that ‘high-risk’ patients are more likely to experience a delay in the receipt of appropriate therapy, increased length of hospital stay, more frequent intensive care unit (ICU) admission, increased cost of care (including antibiotic costs) and increased mortality [4–8].

Treatment guidelines recommend initiation of antibiotic therapy as soon as a patient is diagnosed or suspected of an intra-abdominal infection [3]. Since culture and susceptibility results are not available at diagnosis, empiric antibiotic therapy is often considered. If the initial empiric therapy chosen has in vitro activity against the pathogen isolated it is termed initial appropriate antibiotic therapy (IAAT), whereas one without in vitro activity is termed initial inappropriate empiric therapy (IIAT).

Important considerations for choosing empiric therapy include consideration of the most likely pathogens at the site of infection, knowledge of any prior colonization, and finally, local resistance epidemiology [9–12]. Surgical Infection Society and Infectious Diseases Society of America (IDSA) joint guidelines for treatment of cIAI suggest routine culture and susceptibility studies if there is significant resistance (10–20% of isolates) of a common isolate to an antimicrobial regimen in widespread local use [3]. Improving the chances of IAAT is likely to improve clinical outcomes and impart economic benefits. A US study with cIAI patients identified the additional length of stay (LOS) for IIAT relative to IAAT as 4.6 days (11.6 days vs. 6.9 days total), with additional hospital costs per patient of $6368 ($16,520 vs. $10,152) and substantial excess mortality (9.5% vs. 1.3%) [13].

Given the acute nature of cIAI and the substantial clinical and economic benefits associated with IAAT, the antibacterial spectrum of the empiric antibiotic agent considered should cover the most relevant pathogens to increase the likelihood of IAAT. The economic benefits that could be obtained because of improved susceptibility and increased coverage of IAAT is an important consideration.

A case in point is the comparison of piperacillin/tazobactam and ceftolozane/tazobactam + metronidazole. Piperacillin/tazobactam is recommended for empiric therapy for the treatment of cIAI in various treatment guidelines [14, 15]. Ceftolozane/tazobactam is a novel cephalosporin/β-lactamase inhibitor combination with activity against multi-drug resistant gram-negative pathogens, including extended-spectrum β-lactamase-producing *Enterobacteriaceae* and drug-resistant *P. aeruginosa* [16]. Metronidazole is an oral synthetic antiprotozoal and antibacterial agent which may be used for initial empiric treatment of complicated intra-abdominal infections alongside other agents including ceftolozane/tazobactam. In this study, we assess the cost-effectiveness of ceftolozane/tazobactam + metronidazole compared with piperacillin/tazobactam (considered standard of care) as empiric therapy in the treatment of hospitalized US patients with cIAI.

## Methods

### Model structure

**Fig. 1:**
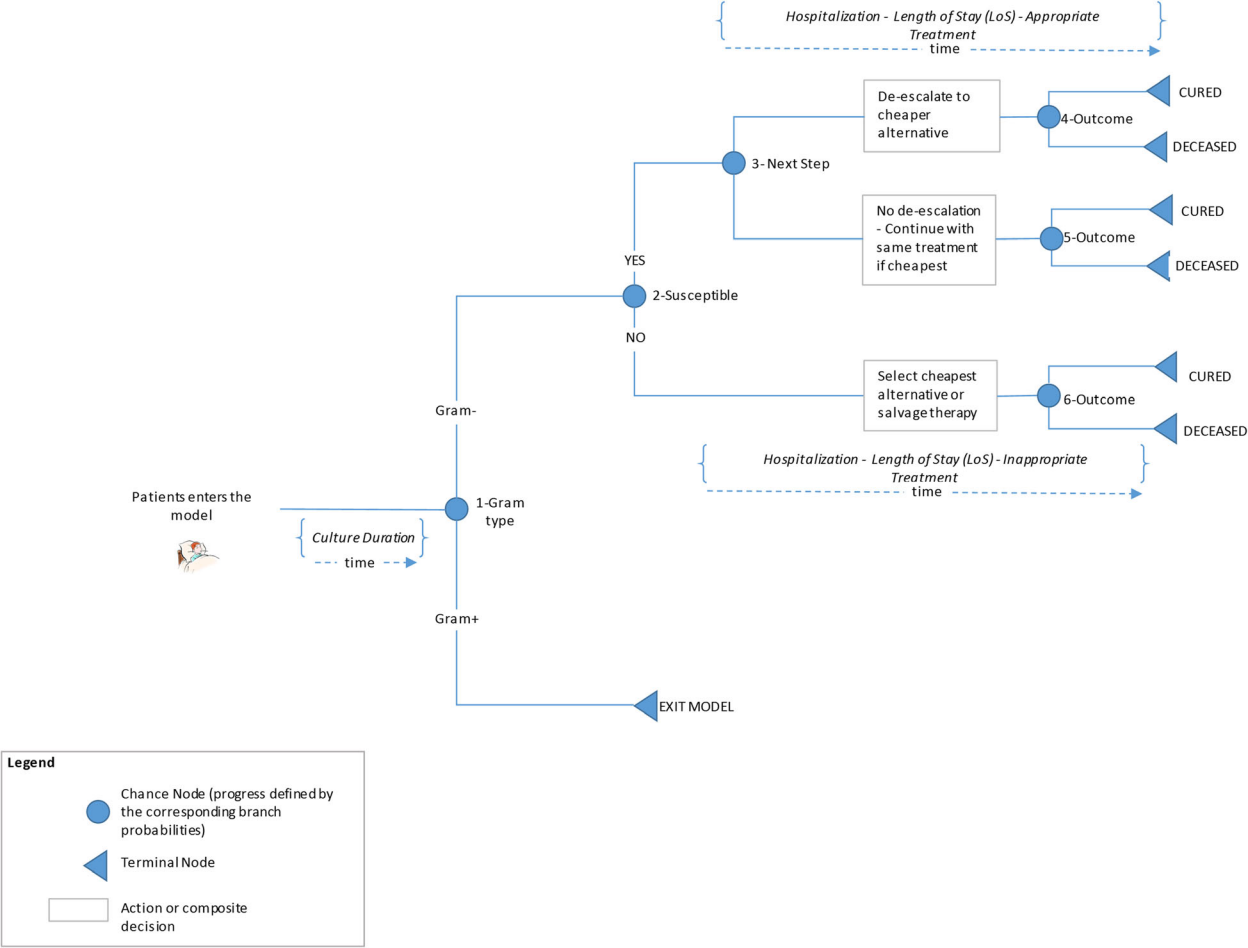
Model structure

We developed a decision-analytic microsimulation model to estimate the quality-adjusted life expectancy and cost of patients admitted to an inpatient facility, diagnosed with cIAI, and administered empiric antibiotic therapy. A graphical representation of the model structure with all treatment pathways is provided in Fig. 1. The methodology and model structure is similar to the one used to assess the cost-effectiveness of ceftolozane/tazobactam in complicated urinary tract infections [17].

Patients enter the microsimulation model at the time of cIAI diagnosis, which is assumed to be concurrent with initiation of empiric antimicrobial therapy. Each patient in the model receives empiric antibiotic treatment with ceftolozane/tazobactam + metronidazole in one arm and piperacillin/tazobactam in another. A specimen is isolated for culture after diagnosis to determine the pathogen and its in-vitro susceptibility to different antibiotic therapies.

Pathogen distribution and in-vitro susceptibility was based on that of a US isolate randomly selected from the Program to Assess Ceftolozane/Tazobactam Susceptibility (PACTS) surveillance dataset, an international antimicrobial surveillance database. Each intra-abdominal pathogen from the PACTS database represents a single patient in the micro-simulation. The types of pathogens can be chosen within the model to allow analyses to be tailored to the underlying pathogens for specific indications. Further details regarding PACTS are provided in Additional file 1.

Treatment pathway and disease progression are estimated using a decision-tree shown in Fig. 1, after the patient is selected. Patients continue empiric treatment until culture results are available. Once culture results are known, patients are switched to the least expensive therapy to which the causative pathogen is susceptible. If the pathogen is not susceptible to any of the modeled comparators, patients are switched to salvage therapy (combination of meropenem and colistin).

The appropriateness of initial antibiotic therapy influences each patient’s length of hospital stay and treatment outcome. Mortality in the model is dependent upon whether the patient experiences IAAT or IIAT (higher mortality rate applied for patients experiencing IIAT).

For patients who survive, we assume that they live a normal length of life based on their life expectancy, and incur health care expenditure comparable to those of the average person their age [18].

Patients with gram-positive pathogens exit the model because they may not be treated by either comparator drugs. We assume that patients incur similar outcomes and costs on either arms if they are gram-positive and therefore economic incremental impact on ceftolozane/tazobactam arm is likely to be negligible.

The model allows us to compute undiscounted and discounted costs and QALYs for each arm, the incremental costs, incremental QALYs and the incremental cost-effectiveness ratio.

### Inputs

#### Susceptibility: Customizing PACTS database to represent cIAI patients

The in-vitro surveillance data from the PACTS database represents the only source of patient-level, real-world data reflecting IAI patients at risk of resistant infection in the US, includes isolate susceptibility to ceftolozane/tazobactam. Isolates obtained from US sites from 2011 to 2013 were included in this analysis. The following organisms were included in line with the approved label for ceftolozane/tazobactam and encompass the major pathogens involved in cIAIs: [2] *Enterobacter cloacae*, *Escherichia coli*, *Klebsiella oxytoca*, *Klebsiella pneumoniae*, *Proteus mirabilis* and *Pseudomonas aeruginosa*.

One limitation of the PACTS database is that it does not differentiate between complicated and uncomplicated IAI. In order to overcome this limitation, isolates in the PACTS database were sampled in proportion to the pathogen distribution for cIAI in a real-world setting found in the Premier hospital discharge database, [19] a complete census of inpatients and hospital-based outpatients from geographically diverse hospitals in the US. More information regarding Premier database is provided in Additional file 1. An algorithm based on a set of ICD-9 diagnosis codes and current procedural terminology (CPT) procedure codes was used to identify cIAI patients from the Premier database between January 1, 2009 and March 31, 2013. The cIAI cohort consisted of 10,159 abdominal isolates, the mean age was 55 ± 22 years (median age: 59 years), and most patients with positive cultures were above 50 years. The resulting pathogen distribution used in the model was 26.6% for *Escherichia coli*, 16.0% for *Klebsiella pneumonia*, 13.5% for *Pseudomonas aeruginosa*, and 9.0% for *Enterobacter cloacae*. The gram-negative pathogens that occurred in less than 5% of patients were grouped together and made up the remaining 5.8%. The percentage of patients with gram-positive pathogens in the cohort was 29.1% [2].

#### Susceptibility breakpoints

The susceptibility is evaluated using Clinical and Laboratory Standards Institute (CLSI) breakpoints. A breakpoint of 2 mg/L was used for *Enterobacteriaceae* and a susceptibility breakpoint of 4 mg/L was used for *Pseudomonas spp.* [20].

#### Drugs used for the model

The empiric treatments used in the model are ceftolozane/tazobactam + metronidazole for one arm and piperacillin/tazobactam for another, which are consistent with the approved therapies and international cIAI treatment guidelines [14, 15]. The following additional drugs were considered for switching upon pathogen confirmation: aztreonam, cefepime, ceftazidime, ceftriaxone, ciprofloxacin, doripenem, imipenem, levofloxacin, meropenem and tigecycline.

#### Clinical inputs

The key clinical inputs are summarized in Table 1. Mortality rates and length of stay were based on Edelsberg et al., where patients who received IIAT spent 4.6 more days in the hospital (11.6 vs. 6.9 total days) [13]. Duration of empiric therapy was assumed to be 3 days. US life-tables were used for the prediction of life expectancy [21]. The percentage of cIAI patients requiring re-intervention has been reported at approximately 8–9% [22, 23]. While most published studies examining the impact of IIAT on treatment outcomes in cIAI did not report re-intervention rates, there is evidence from at least one study that IIAT may increase the risk of re-intervention (relative risk ratio, 5.1; 95% CI, 1.7–15.4) [24]. As the Krobot et al. study [24] was relatively small and conducted over a decade ago, the analysis conservatively assumed that there was no differential impact of IIAT on re-intervention. Similarly, any costs associated with re-intervention, such as imaging, were excluded from the model since those costs did not vary by empiric treatment option.

**Table 1:** Clinical and economic inputs

Input Parameters	Mean	Lower bound	Upper bound	Source
Mortality rate with appropriate empiric treatment	0.013	0.012	0.014	Edelsberg et al. [13]
Mortality rate with inappropriate empiric antibiotic	0.095	0.086	0.105	Edelsberg et al. [13]
Duration of empiric therapy	3 days	3 days	3 days	Assumption
Total LOS for IAAT (inc. empiric therapy)	6.9 days	6.8 days	7 days	Edelsberg et al. [13]
Total LOS for IIAT (inc. empiric therapy)	11.5 days	11.3 days	11.9 days	Edelsberg et al. [13]
Health utility for survivors	0.85	0.70	1.00	Assumption based on Jansen et al. [25]
Discount rate	3.0%	3.0%	3.0%	AMCP [26]
Hospital cost per day (average)	$2558.55	$2046.84	$3070.26	HCUP [27]
Drug acquisition costs per day				
Ceftolozane/tazobactam plus metronidazole	$253.20			Analy$ource [29]
Aztreonam	$84.24			Analy$ource [29]
Cefepime	$23.04			Analy$ource [29]
Ceftazidime	$36.66			Analy$ource [29]
Ceftriaxone	$6.40			Analy$ource [29]
Ciprofloxacin	$5.26			Analy$ource [29]
Doripenem	$125.22			Analy$ource [29]
Imipenem	$73.12			Analy$ource [29]
Levofloxacin	$6.24			Analy$ource [29]
Meropenem	$81.51			Analy$ource [29]
Piperacillin/tazobactam	$43.08			Analy$ource [29]
Tigecycline	$238.34			Analy$ource [29]
Salvage^a^	$164.31			Analy$ource [29]
Health care expenditure incurred per year				
<25 years	$477			Basu [18]
25 to 34 years	$790			Basu [18]
35 to 44 years	$947			Basu [18]
45 to 54 years	$1422			Basu [18]
55 to 64 years	$2106			Basu [18]
65 to 74 years	$2758			Basu [18]
75 years and above	$3100			Basu [18]

^a^Salvage therapy consists of meropenem + colistin for cost purposes

*LOS* Length of stay, *IAAT* Initial appropriate antibiotic therapy, *IIAT* Initial inappropriate antibiotic therapy

An assumed utility value of 0.85 was applied to cured patients for the remainder of their lives (Table 1). This was a conservative estimate based on a utility value of 0.9 proposed by Jansen et al. [25]. QALYs were discounted at a rate of 3% per annum [26].

#### Economic inputs

Hospitalization costs per day (Table 1) were derived from the 2013 Healthcare Cost and Utilization Project (HCUP) [27] and inflated to 2015 values using the Gross Domestic Product (GDP) price index [28]. Hospitalization costs were based on primary diagnoses for cIAI (ICD-9 code 540.0, 540.1, 567.0, 567.21, 567.22, 567.23, 567.29, 567.31, 567.89, 567.9, and 569.5) [27]. The average cost per hospital day for cIAI patients, inflated to 2015 values, was $2558.55.

Daily drug costs (Table 1) were calculated for the duration of hospitalization based on wholesale acquisition cost at labeled doses [29].

For healthy survivors, lifetime health care expenditure was calculated using average annual age-adjusted values [18] inflated to 2015 values using the Gross Domestic Product (GDP) price index (Table 1) [28].

Hospitalization and daily drug costs were not discounted as all costs were incurred within the first year, given the acute nature of cIAI. A discount rate of 3% per annum was applied to lifetime health care expenditure for health survivors.

### Analysis

A lifetime time horizon was applied to capture the costs and utility of healthy survivors over their lifetime. The model compared ceftolozane/tazobactam + metronidazole with piperacillin/tazobactam from the healthcare perspective.

To compare the two treatment strategies the following outcomes were estimated from the model: proportion of patients appropriately and inappropriately treated (sensitive/resistant to empiric therapy, cost per QALY saved, drug costs, hospitalization costs, proportion of cases by pathogen, total costs, total QALYs). Differences in these outcomes of interest were estimated, along with the incremental cost-effectiveness ratio (ICER) based on total cost per QALY gained.

One-way sensitivity analyses (OWSA) and probabilistic sensitivity analysis (PSA) were performed to quantify the uncertainty in the model outcomes based on the uncertainty of the input parameters. The model assessed the sensitivity of the model results to all the input data for which uncertainty has been defined one parameter at a time by means of OWSA. The parameters with the greatest impact were summarized with tornado diagrams.

Ten thousand samples were taken to estimate ranges for the PSA. Input parameter values were sampled from the defined distributions for efficacy, safety, and costs. Lognormal distributions were used for odds ratios, beta distributions for utilities, and for gamma distributions for resource use and costs.

For each treatment strategy, the probability of cost-effectiveness was expressed with cost-effectiveness acceptability curves, calculated as the number of iterations out of the total number of iterations for which the net monetary benefit (NMB) was greatest for a given treatment strategy out of all strategies.

The NMB was calculated as the QALYs multiplied by a willingness to pay (WTP) ratio minus the costs, where the WTP is the amount decision makers were willing to pay per additional QALY gained. An amount of US $100,000 was used as a WTP threshold [30].

Risk factors associated with infection due to resistant pathogens (vs. susceptible pathogens) have been identified in the literature [31, 32]. Information regarding a portion of these risk factors for cIAI was available for patients in the PACTS dataset, including (a) nosocomial infection, (b) age ≥ 65 years, and (c) ICU stay.

Scenario analyses were performed firstly using all available isolates for high risk patients aged ≥65 years and requiring an ICU stay, and secondly using only nosocomial isolates for high risk patients aged ≥65 years and requiring an ICU stay.

An additional scenario was also performed excluding lifetime health care expenditure for healthy survivors.

## Results

### Base case results

In the cohort of 1000 patients, the average age was 67.1 years ranging from 21 to 100 years.

The key results from the model are summarized in Table 2. Under the base case scenario, ceftolozane/tazobactam + metronidazole arm resulted in lower total costs than the piperacillin/tazobactam arm ($44,226 per patient vs. $44,811). The ceftolozane/tazobactam + metronidazole arm also experienced a greater number of QALYs than the piperacillin/tazobactam arm (12.85 per patient vs. 12.70 per patient). This resulted in ceftolozane/tazobactam + metronidazole dominating piperacillin/tazobactam with 0.63 hospitalization days saved per patient.

**Table 2:** Results

Summary of results	Ceftolozane/tazobactam + metronidazole	Piperacillin/tazobactam	Incremental Ceftolozane/tazobactam + metronidazole - Piperacillin/tazobactam
Total costs per patient	$44,226	$44,811	-$585
Total life years per patient (undiscounted)	21.75	21.50	0.25
Total QALYs per patient (undiscounted)	18.49	18.27	0.22
Total QALYs per patient (discounted)	12.85	12.70	0.15
Incremental Cost Effectiveness Ratio (Cost per discounted QALY saved)	–	–	Dominant
Hospitalization days saved per-patient	–	–	0.63
Distribution of patients based on empiric treatment			
Resistant to initial therapy (%)	35.2	48.8	–
Susceptible to initial therapy (%)	64.8	51.2	–
Costs			
Hospital costs per patient	$15,468	$17,069	-$1601
Drug costs per patient	$818	$196	$622
Lifetime health care expenditure per patient	$27,940	$27,546	$394

*QALY* Quality Adjusted Life Year, *IAAT* Initial appropriate antibiotic therapy, *IIAT* Initial inappropriate antibiotic therapy

In patients with a gram-negative infection receiving ceftolozane/tazobactam + metronidazole as empiric therapy, 6.1% were resistant compared with 19.7% in patients receiving piperacillin/tazobactam. Since 29.1% of pathogens were gram-positive, overall, 35.2% were not susceptible to ceftolozane/tazobactam + metronidazole compared with 48.8% for piperacillin/tazobactam. There were 41.8 deaths (4.2%) in the ceftolozane/tazobactam + metronidazole arm compared with 53.0 (5.3%) in the piperacillin/tazobactam arm. Amongst those who died, a larger proportion was resistant to initial therapy in the piperacillin/tazobactam arm than the ceftolozane/tazobactam + metronidazole arm. Ceftolozane/tazobactam + metronidazole reduced overall mortality by 1.1% versus piperacillin/tazobactam.

When examining the QALY results in more detail, the ceftolozane/tazobactam + metronidazole arm generated 0.15 more QALYs (discounted) per patient. The average number of QALYs (discounted) experienced by patients in the ceftolozane/tazobactam + metronidazole arm were 12.85 and 12.70 for ceftolozane/tazobactam + metronidazole and piperacillin/tazobactam, respectively.

Lifetime health care expenditure was the largest contributor to total costs in both treatment arms followed by hospital costs. The average lifetime health care expenditure per patient in the ceftolozane/tazobactam + metronidazole arm was higher than in the piperacillin/tazobactam arm ($27,940 vs. $27,546). The average hospital cost per patient in the ceftolozane/tazobactam + metronidazole arm was lower than in the piperacillin/tazobactam arm ($15,468 vs. $17,069, respectively). Per-patient drug costs in the ceftolozane/tazobactam + metronidazole arm were slightly higher than in the piperacillin/tazobactam arm ($818 vs. $196, respectively).

All of the patients in the ceftolozane/tazobactam + metronidazole arm who received IAAT were able to switch to a less expensive therapy after 3 days (following culture results). For 1.0% of patients in the piperacillin/tazobactam arm who received IAAT, empiric therapy with piperacillin/tazobactam was the least expensive treatment option. In patients who received IIAT, an equal number of patients in each arm (*n* = 19, 1.9%) required salvage therapy with meropenem + colistin.

### One-way sensitivity analysis

**Fig. 2:**
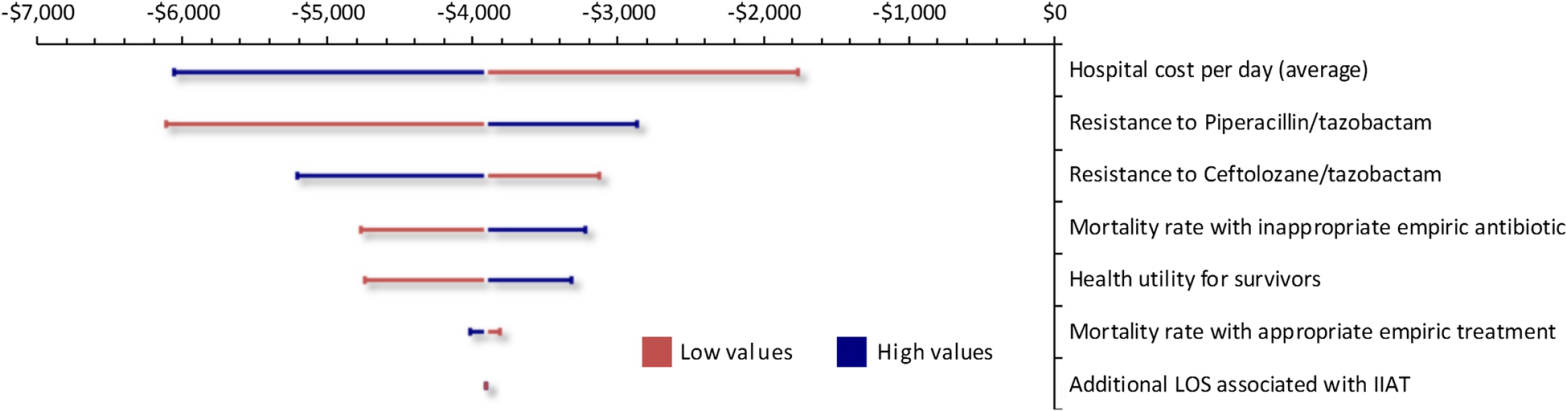
Ceftolozane/tazobactam vs. piperacillin/tazobactam: influence of variables on ICER (cost per discounted QALY). ICER = Incremental cost-effectiveness ratio; QALY = Quality Adjusted Life Year

The results of the one-way sensitivity analysis are presented in a tornado graph (Fig. 2). Varying the average cost per hospital day resulted in the largest impact on the resultant ICER. The other input parameters which impacted the model results when varied were: resistance to piperacillin/tazobactam, resistance to ceftolozane/tazobactam, mortality rate with IIAT, the utility value applied to survivors, and the mortality rate with IAAT. Varying the additional length of stay associated with IIAT had very little impact on the ICER. In all instances, ceftolozane/tazobactam + metronidazole remained the dominant option versus piperacillin/tazobactam.

### Probabilistic sensitivity analysis

The distribution of ICER estimates from the PSA show that in all instances, ceftolozane/tazobactam + metronidazole is more effective and less costly than piperacillin/tazobactam.

Subsequently, ceftolozane/tazobactam has a 100% probability of being cost-effective compared with piperacillin/tazobactam at a willingness-to-pay threshold of $100,000/QALY gained.

### Scenario analyses

Seventy-four percent of patients from the US PACTS dataset were aged ≥65 years and 4% of patients required an ICU stay. Thirty-eight percent of patients had isolates from nosocomial sources. Amongst these patients, 37% were aged ≥65 years and 5% of patients required an ICU stay. Patients could be associated with more than one risk factor.

Results of the scenario analyses are presented in Table 3. Overall, the cost-effectiveness of ceftolozane/tazobactam + metronidazole improves versus piperacillin/tazobactam in high risk patients (≥65 years and requiring an ICU stay) and high risk patients with nosocomial infections. Differences in costs and the number of hospitalization days are larger in both of the subgroups explored, with a larger difference in total QALYs (discounted) seen in the high risk patients with nosocomial infections.

**Table 3:** Scenario analyses results

Results for high risk patients (aged 65 years and requiring an ICU stay) using all available isolates	Ceftolozane/tazobactam + metronidazole	Piperacillin/tazobactam	Incremental Ceftolozane/tazobactam + metronidazole - Piperacillin/tazobactam
Total costs per patient	$41,838	$42,501	-$662
Total QALYs (discounted) per patient	11.38	11.24	0.14
Incremental Cost Effectiveness Ratio (Cost per discounted QALY saved)	–	–	Dominant
Results for high risk patients (aged 65 years and requiring an ICU stay) using nosocomial isolates	Ceftolozane/tazobactam + metronidazole	Piperacillin/tazobactam	Incremental Ceftolozane/tazobactam + metronidazole - Piperacillin/tazobactam
Total costs per patient	$42,979	$44,403	-$1424
Total QALYs (discounted) per patient	11.75	11.53	0.22
Incremental Cost Effectiveness Ratio (Cost per discounted QALY saved)	–	–	Dominant
Results when lifetime health care expenditure for health survivors is excluded	Ceftolozane/tazobactam + metronidazole	Piperacillin/tazobactam	Incremental Ceftolozane/tazobactam + metronidazole - Piperacillin/tazobactam
Total costs per patient	$16,286	$17,265	-$978
Total QALYs (discounted) per patient	12.85	12.70	0.15
Incremental Cost Effectiveness Ratio (Cost per discounted QALY saved)			Dominant

QALY Quality Adjusted Life Year

In the scenario where lifetime health care expenditure was excluded, the total costs per patient were considerably lower and the incremental cost between ceftolozane/tazobactam + metronidazole and piperacillin/tazobactam was larger. Ceftolozane/tazobactam + metronidazole remained the dominant choice.

## Discussion

The objective of this analysis was to evaluate the use of ceftolozane/tazobactam + metronidazole compared with piperacillin/tazobactam in the empiric treatment of US patients with cIAI at risk of infection due to a resistant gram-negative pathogen. The ability of either ceftolozane/tazobactam + metronidazole or piperacillin/tazobactam to provide appropriate empiric coverage is an important concept in the model and the source of economic differentiation between the two therapies. Ceftolozane/tazobactam + metronidazole provides a greater degree of appropriate empiric coverage than piperacillin/tazobactam, as demonstrated by the PACTS data.

We have presented a novel approach utilizing surveillance data to evaluate the cost-effectiveness of two empiric therapy options. A similar approach to ours was used in the study by Sader et al., where they used the SENTRY Antimicrobial Surveillance Program, a large multinational data source on pathogen prevalence and antimicrobial susceptibility, to estimate the effectiveness of tigecycline in complicated skin and skin structure infections [33]. Although this study only considered effectiveness and not costs.

The findings of our analysis suggest that the use of ceftolozane/tazobactam + metronidazole as initial (empiric) treatment may result in substantial cost-savings compared to piperacillin/tazobactam. Additionally, use of ceftolozane/tazobactam + metronidazole may save an average of 0.63 hospital days per patient.

IIAT is a key driver to the model and contributes to the differentiation between ceftolozane/tazobactam + metronidazole and piperacillin/tazobactam. The impact of IIAT is further emphasized in our two high risk scenarios. In both scenarios, susceptibility rates to ceftolozane/tazobactam + metronidazole remain largely unchanged, however, susceptibility rates to piperacillin/tazobactam are lower compared to the base case.

Cost savings are a function of several model parameters including duration of empiric therapy, susceptibility among comparators, and the increase in length of stay due to IIAT. Furthermore, differences in costs derive solely from differences in antimicrobial activity between ceftolozane/tazobactam + metronidazole and piperacillin/tazobactam.

The inclusion of lifetime health care expenditures in our base case analysis reduced the incremental costs by approximately 50%. For our analysis, ceftolozane/tazobactam + metronidazole remained the dominant option, however, inclusion of lifetime healthcare expenditure may have a potential impact on comparisons which are borderline cost-effective or cost-saving.

We have shown how data from national surveillance data set can be used to guide the choice of cost-effective empirical therapy. Clinical trials are often conducted in a variety of different geographic locations/settings and the patients enrolled may not necessarily reflect the specific populations who will receive these treatments in real life. In practice, patient outcomes can be improved through improvements in the collection of local surveillance data and the use of local antibiograms in decision making and guideline development.

## Limitations

An important limitation is that the model does not account for further treatment changes after any initial de-escalation/escalation, with patients assumed to be fully cured or dead at the end of hospitalization.

Additionally, recurrence and/or re-admission were not incorporated in this model. For readmission rates, we assumed that these were the same for patients with IAAT and IIAT, and subsequently did not have any economic impact. If patients with IIAT have a higher rate of readmission, the subsequent analysis would further improve results favoring the ceftolozane/tazobactam arm.

The model assumes that the duration of therapy, whilst shorter for IAAT compared with IIAT, is not directly impacted by the different drugs used following culture results. In practice, some treatments may shorten/prolong hospital length of stay.

The PACTS dataset was not designed to focus on resistant or complicated IAI. Therefore it did not contain enough information to specifically target complicated IAI (vs. uncomplicated IAI) patients and may under-represent pathogen resistance in the target population of cIAI. We attempted to overcome this limitation by sampling isolates in the PACTS database in proportion to the pathogen distribution for cIAI in a real-world setting found in the Premier hospital discharge database. PACTS is the only source of patient level, real-world data reflecting IAI patients at risk of resistant infection in the US that includes isolate susceptibility to ceftolozane/tazobactam. Our sample had 294 isolates from patients with IAIs. To conduct an analysis representative of local settings, more data at a local level may be needed. Also, within the PACTS database, only one isolate per patient infection was included in the surveillance whereas in clinical practice you are likely to encounter more than one isolate per patient.

Additional limitations are the exclusion from the model of bacterial resistance over time and costs of antibiotic preparation and administration, monitoring, and adverse events. These costs were assumed to be similar across treatments and/or minor. Similarly, dose adjustments were not considered.

The model only considers gram-negative aerobes, when in practice, gram-positive aerobes and anaerobes (both gram-positive and gram-negative) are frequently implicated. The proportion of patients with gram-positive infections in our cohort was based on the distribution of gram-positive bacteria identified from intraoperative samples reported by Sartelli et al. [2]. This figure may not be entirely accurate due to the fact that patients can harbor more than one type of bacteria, affecting that actual distribution of gram-positive bacteria amongst patients.

## Conclusion

Economic models utilizing surveillance data can help to identify the appropriate choice of empiric therapy for the treatment of cIAI. The results of this cost-effectiveness model indicate that cost-savings and improvements in QALYs may be achieved by the empiric use of ceftolozane/tazobactam + metronidazole instead of piperacillin/tazobactam in US cIAI patients at risk of resistant infection.

## Additional file

Additional file 1: Susceptibility inputs - Program to Assess Ceftolozane/Tazobactam Susceptibility (PACTS) dataset. (DOCX 25 kb)

## Abbreviations

cIAI: complicated intra-abdominal infection
CLSI: Clinical and Laboratory Standards Institute
CPT: Current procedural terminology
GDP: Gross Domestic Product
HCUP: Healthcare Cost and Utilization Project
IAAT: Initial appropriate antibiotic therapy
ICD-9: International Classification of Diseases, Ninth Edition
ICER: Incremental cost-effectivness ratio
ICU: Intensive care unit
IDSA: Infectious Diseases Society of America
IIAT: Initial inappropriate antibiotic therapy
LOS: Length of stay
NMB: Net monetary benefit
PACTS: Program to Assess Ceftolozane/Tazobactam Susceptibility
QALYs: Quality-adjusted life years
US: United States
WTP: Willingness to pay
