# Supplementary material for: Cost-effectiveness of ceftolozane/tazobactam plus metronidazole versus piperacillin/tazobactam as initial empiric therapy for the treatment of complicated intra-abdominal infections based on pathogen distributions drawn from national surveillance data in the United States
Source: Antimicrob Resist Infect Control. 2017 Oct 27;6:107. doi: 10.1186/s13756-017-0264-2 (PMC5658949; doi:10.1186/s13756-017-0264-2)
Supplement: Additional file 1: — Susceptibility inputs - Program to Assess Ceftolozane/Tazobactam Susceptibility (PACTS) dataset. (DOCX 25 kb) [file 13756_2017_264_MOESM1_ESM.docx]

**Additional file 1**

Susceptibility inputs - Program to Assess Ceftolozane/Tazobactam Susceptibility (PACTS) dataset

The PACTS is a surveillance study evaluating the activity of ceftolozane/tazobactam and numerous Gram-negative agents against contemporary clinical isolates obtained from hospitalized patients with serious infections including bloodstream infections, skin and skin structure infections, urinary tract infections, intra-abdominal infections, and respiratory tract infections. The isolates were consecutively collected from 28 medical centers located in the US and 31 medical centers in 15 European countries from January 2011 to December 2013.[1] One isolate per patient infection was included in the surveillance.

In order to assess the antimicrobial activity of ceftolozane/tazobactam and other (comparator) agents, the isolates were tested for susceptibility to these agents. Minimum inhibitory concentration (MIC) values of the agents were determined using standard broth micro-dilution methods as described by the Clinical and Laboratory Standards Institute (CLSI).[2] MIC values at 90% were interpreted according to susceptible and resistant breakpoints for comparator antibiotics.

Complicated intra-abdominal infection pathogen distribution – Premier database

Premier is an alliance of community-based hospitals with over 2,800 hospital members.[3] The database is a complete census of inpatients and hospital-based outpatients from geographically diverse hospitals in the US. The database currently contains data from more than 480 million patient encounters, or one in every five discharges in the US. Laboratory results are available from a subset of facilities in the Premier research database.

References

1. Sader HS, Farrell DJ, Flamm RK, Jones RN: **Ceftolozane/tazobactam activity tested against aerobic Gram-negative organisms isolated from intra-abdominal and urinary tract infections in European and United States hospitals (2012)**. *The Journal of infection* 2014, **69**(3):266-277.

2. Clinical and Laboratory Standards Institute: **Performance Standards for Antimicrobial Susceptibility Testing. CLSI supplement M100S.**, 26th edn; 2016.

3. Ready NK, N. A. Orlov, S. V. Luft, A. V. Popovych, O. Holmlund, J. T. Wood, B. A. Leopold, L.: **Double-blind, placebo-controlled, randomized phase 2 study of the proapoptotic agent AT-101 plus docetaxel, in second-line non-small cell lung cancer**. *Journal of Thoracic Oncology* 2011, **6**(4):781-785.
